# The Relationship Between rs3212986C>A Polymorphism and Tumor Stage in Lung Cancer Patients

**DOI:** 10.7759/cureus.4423

**Published:** 2019-04-10

**Authors:** Ali Arash Anoushirvani, Reza Aghabozorgi, Azam Ahmadi, Mohammad Arjomandzadegan, Sara Khalili, Maryam Sahraei, Taha Fereydouni, Zoha Khademi

**Affiliations:** 1 Internal Medicine, Arak University of Medical Sciences, Arak, IRN; 2 Genetics, Arak University of Medical Sciences, Arak, IRN; 3 Miscellaneous, Arak University of Medical Sciences, Arak, IRN; 4 Microbiology, Arak University of Medical Sciences, Arak, IRN

**Keywords:** nsclc, ercc1, sequencing, clinicopathological features

## Abstract

Background

The nucleotide excision repair (NER) system is one of the most important deoxyribonucleic acid (DNA) repair mechanisms and is critical for chemotherapy resistance. We conducted the present study to investigate the association between two polymorphisms of excision of repair cross-complementing group 1 (ERCC1), the key component of the NER pathway, and the clinicopathological features of patients with non-small cell lung cancer (NSCLC).

Methods

A total of 38 patients with confirmed NSCLC were included in our study. DNA was extracted from peripheral blood. ERCC1 rs3212986 (8092) and rs11615 (118) were genotyped using molecular assays including polymerase chain reaction (PCR) with restriction fragment length polymorphism (by MboII and HpyCH4 enzymes) and sequencing.

Results

The PCR results indicated the correct performance of the genomics extraction and molecular protocols. The distribution of C/C, C/A and A/A genotypes at position 8092 was 42.10%, 47.36%, and 10.52% respectively (P=0.03). Multivariate regression analysis showed that there was a significant correlation between C8092A (rs3212986) polymorphism and metastasis, grade of the tumor, and response to treatment. Individuals carrying the rs3212986 CA genotype and A allele had a significantly worse response to the treatment. Also, the correlation between alteration at this genomics location and patients with NSCLC who used to smoke cigarettes was positive. However, no significant association was detected between rs11615 C118>T polymorphism and demographic characteristics of patients with NSCLC.

Conclusion

We concluded that in lung cancer patients there is a relationship between tumor stage and rs3212986C>A polymorphism.

## Introduction

Every year, 1.35 million new cases and 1.18 million deaths are reported due to lung cancer [1]. Based on the cells affected by transformation, there are various forms of lung cancer; each form has its own symptoms and complications [2]. Although the genetic cause of this disease has not been identified properly, its relationship with the occurrence of changes in several genes has been proven. The products of each of these genes exist in different signaling pathways. Specialists have classified lung cancer into small cell lung cancer (SCLC), and non-small cell lung cancer (NSCLC) types according to the difference in their treatment [3]. Despite recent advances in cancer treatment, the survival rate for patients with lung cancer is very low, so its diagnosis by precise molecular methods is necessary [4]. One cause of cancer is the presence of defects in the genomics repair systems. One of the more important of these systems is the Nucleotide Excision Repair (NER) system [5-6]. Thymine dimers and large chemical complexes are the main targets of this system and are identified by this system. Repair by nucleotide extraction in eukaryotes and prokaryotes are largely similar; the only significant difference between these two methods is the number of removed nucleotides and involved enzyme complexes. One of the genes involved in the deoxyribonucleic acid (DNA) repair system by the NER method is excision repair cross-complementing group 1 (*ERCC1*) [7]. In the NER pathway, the product of the *ERCC1* gene is necessary to repair DNA damages. The heterodimeric endonuclease *ERCC1* mediates the incision on the 5-prime direction in the NER process. In addition, *ERCC1* is involved in cross-link repair during the recombination process. Polymorphism of this gene plays a role in the carcinogenesis process. Polymorphism of this gene has been studied in many types of cancer in different populations [8-10]. The existence of single nucleotide polymorphisms (SNPs) in the key gene of the DNA repair system in breast cancer was identified in a study in 2018 [11]. A study in Japan in 2013 investigated polymorphism in three genomic regions including the *ERCC1* gene in colorectal cancer [8]. In numerous studies, mutation and polymorphism in the hot-spots of the *ERCC1* gene in relation to the NER system have been mentioned in other cancers including glioma, cervix, and bladder in different populations [12-14]. This gene has a length of 71,534 base pairs and many transcript variants. Codon 118 and nucleotide 8092 are two of the major areas susceptible to alteration in this gene.

This study evaluated the existence of polymorphism in tumor samples in relation to the clinical features of the affected population. These data will be important for the timely diagnosis of cancer and attaining the diagnostic biomarkers of lung cancer in early stages.

The aim of this study was to investigate polymorphism in the mentioned areas, converting nucleotide C to A at position 8092 (rs3212986) and exchanging nucleotide C with T at codon 118 (rs11615) in the *ERCC1* gene in patients with lung cancer.

## Materials and methods

Sample collection

The clinical sample collection procedure was approved by the ethics committee of Arak University of Medical Science, Arak, Iran (IR.ARAKMU.REC.1396.7). A total of 38 blood samples from patients with lung cancer was obtained from Khansari Hospital, Arak, Iran.

Polymerase chain reaction

DNA was extracted using PZP Molecular IVD (Iran) according to the manufacturer’s instructions. We used 50 ng of Genomic DNA for polymerase chain reaction (PCR) using Taq DNA Polymerase Master Mix Red (Ampliqon) in a thermal cycler machine (Eppendorf, Germany). The primer sequences [10] used in the amplification reaction are presented in Table 1.

**Table 1:** Primer sequences used in this study

Primer ID	Sequence (5'-3')	Product size
ERCC1 118-F	GCAGAGCTCACCTGAGGAAC	200 bp
ERCC1 118-R	GAGGTGCAAGAAGAGGTGGA	
ERCC1 8092-F	TGAGCCAATTCAGCCACTAGAG	255 bp
ERCC1 8092-R	CTTTAGTTCCTCAGTTTCCCG	

For negative control, a sample without genomic DNA was used. We used a horizontal electrophoresis system to evaluate the accuracy of the produced amplicons after the reaction at an annealing temperature of 59.9°C.

Restriction fragment of length polymorphism and sequencing

To investigate the occurrence of mutations in nucleotides 8092 and 118 of the ERCC1 gene, the amplicons produced were digested by MboII and HpyCH4 enzymes (Fermentas) at 37°C for three hours for restriction fragment of length polymorphism (RFLP) and sequencing. The inactivation of these reactions was carried out at 65°C. Some amplicons were sent for sequencing with the ABI Applied Biosystem-Model 3730XL device (Macrogen CO., South Korea).

Statistics analysis

Data were analyzed using Chromas, Mega, EditSeq, BLAT, and an HWE test calculator. Conservation was evaluated via the UCSC Genome Browser. The difference between the groups was compared by one-way analysis of variance test using GraphPad Prism software version 7.0. The P values < 0.05 were considered statistically significant.

## Results

In this case-control study, 38 lung cancer and 38 control samples were obtained with coordination and correspondence. Patient and control ages ranged from 33 to 75 years (Table 2).

**Table 2:** Characteristics of collected samples and relation with haplotypes of 8092 NSCLC, non-small cell lung cancer.

Characteristic	Detail	No. of patients n(%)		CC n(%)		CA n(%)		AA n(%)		(CA & AA) n(%)	
Total number of patients	NSCLC	38	100	16	42.10	18	47.36	4	10.52	22	57.89
Median age, range	≤58	18	48	8	21.05	8	21.05	2	5.26	10	26.31
	>58	20	52	9	23.68	9	23.68	2	5.26	11	28.94
Gender	Male	30	80	12	31.57	15	39.47	3	7.89	18	47.36
	Female	8	20	5	13.15	2	5.26	1	2.63	3	7.89
T stage	1	6	16	5	13.15	0	0	1	2.63	1	2.63
	2	9	24	4	10.52	3	7.89	2	5.26	5	13.15
	3	11	28	2	5.26	8	21.05	1	2.63	9	23.68
	4	12	32	3	7.89	8	21.05	1	2.63	9	23.68
N stage	0 and 1	12	32	11	28.94	0	0	1	2.63	1	2.63
	2	8	20	4	10.52	3	7.89	1	2.63	4	10.52
	3	18	48	2	5.26	15	39.47	1	2.63	16	42.10
Metastasis	Yes	24	64	10	26.31	12	31.57	2	5.26	14	36.84
	No	14	36	7	18.42	6	15.78	1	2.63	7	18.42
Response to therapy	Partial	5	12	0	0	5	13.15	0	0	5	13.15
	Stable & progressive	21	56	7	18.42	13	34.21	1	2.63	14	36.84
	Non-evaluable	12	32	5	13.15	6	15.78	1	2.63	7	18.42
Smoker	Yes	9	24	3	7.89	5	13.15	1	2.63	6	15.78
	No	29	76	14	36.84	12	31.57	3	7.89	15	39.47
Total number of control group	control (non-patient)	38	100	8	21.05	22	57.89	8	21.05	30	78.94

PCR and RFLPS results

**Figure 1:**
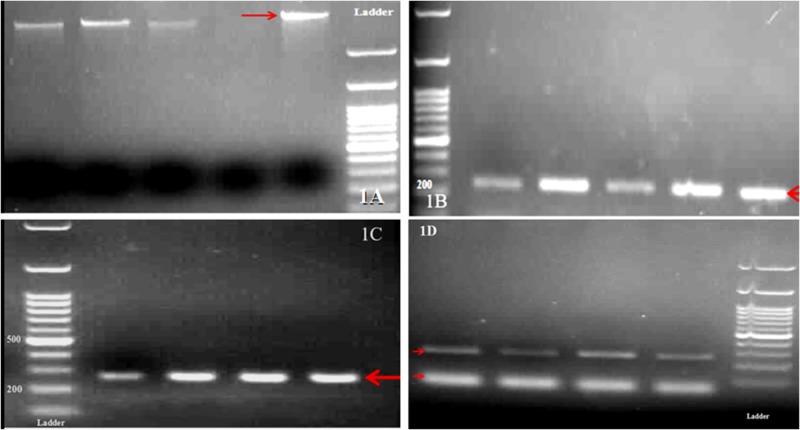
A. Extracted DNA from clinical samples.B. Amplicons associated with codon 118 of the ERCC1 gene. 1C. Amplicons associated with position 8092 of the ERCC1 gene. 1D. Products from the enzymatic digestive reaction. Digestion with MboII created 158bp (C/C), 41-117-158bp (C/A), and 41-117 bp (AA) at 8092 of ERCC1

The PCR-RFLPS results showed the accuracy of primer designing and performance of amplification reaction (Figures 1A, 1B, 1C, 1D).

ERCC1 sequencing result

**Figure 2:**
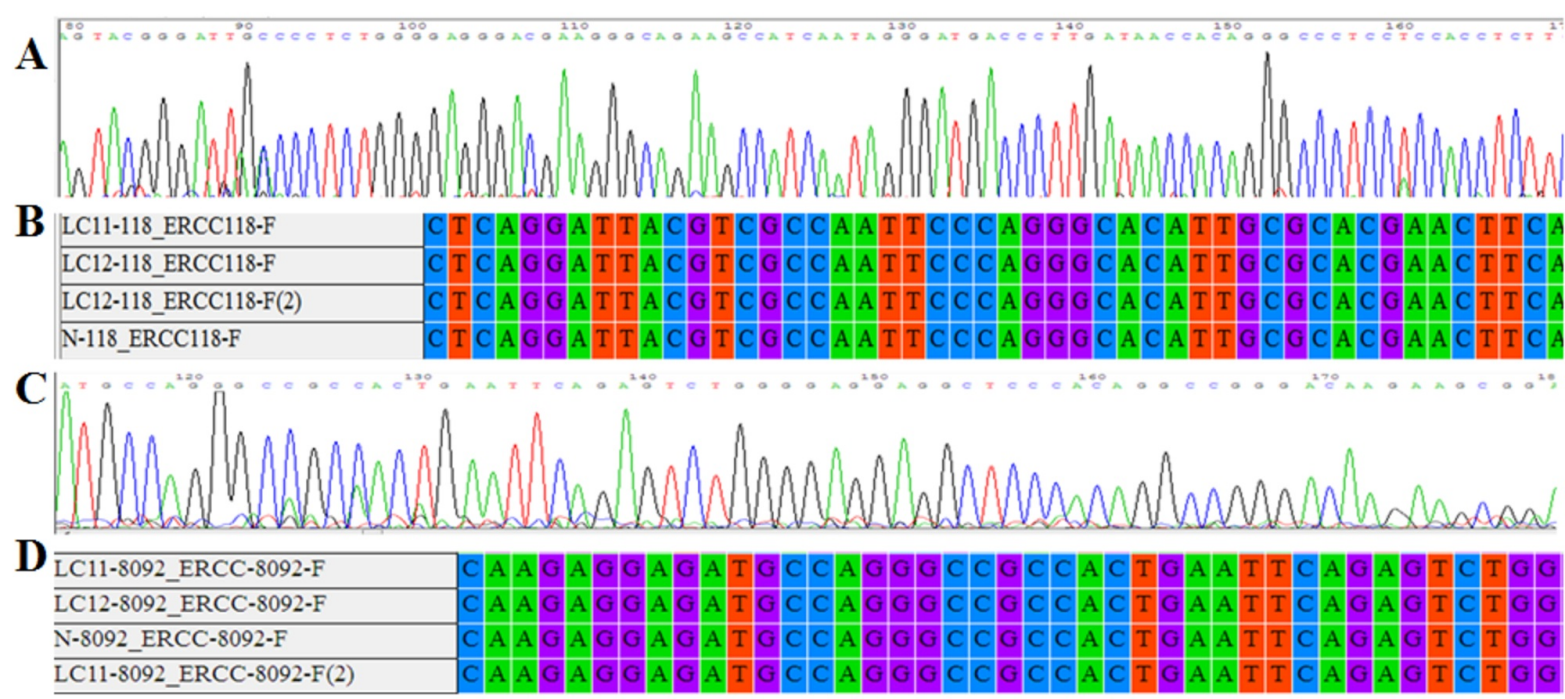
A, B. A screenshot of the CHROMAS and MEGA4 software associated with the sequences of codon 118 of the ERCC1 gene.C,D. A screenshot of the CHROMAS and MEGA4 software related to the 8092 position of the ERCC1 gene

The sequences were evaluated using various software (Figure 2).

The distribution of C/C, C/A, and A/A genotypes at the 8092 position in the patient group was 42.10%, 47.36%, and 10.52%, respectively (P=0.03) (Table 3).

**Table 3:** Patterns from digestion reaction using MboII enzyme OR, odds ratio.

Row	Pattern from digestion reaction	Band (bp)	Genotype	Patients (%)	OR	95% confidence interval	P value
1	Template A	158	CC homozygote	42.10	2.65	1.43 to 4.92	P < 0.05
2	Template B	158, 41, 117	CA heterozygote	47.36	0.67	0.38 to 1.16	P < 0.05
3	Template C	41,117	AA homozygote	10.52	0.41	0.95 to 0.18	P < 0.05

This distribution of the control group at 8092 was 21.05, 57.89, and 21.05. However, no significant association was detected between rs11615 C118>T polymorphism and demographic characteristics of NSCLC. In the patient group at the 8092 position of the ERCC1 gene, the C allele frequency was 0.66 and the A allele frequency was 0.34 (Hardy-Weinberg Equilibrium calculator: Chi-square=0.48693, P<0.05). These frequencies in the control group were 0.46 and 0.54, respectively. The relationship between the clinicopathological properties of clinical samples and the occurrence of mutations in the involved hot-spots of the ERCC1 gene.

**Figure 3:**
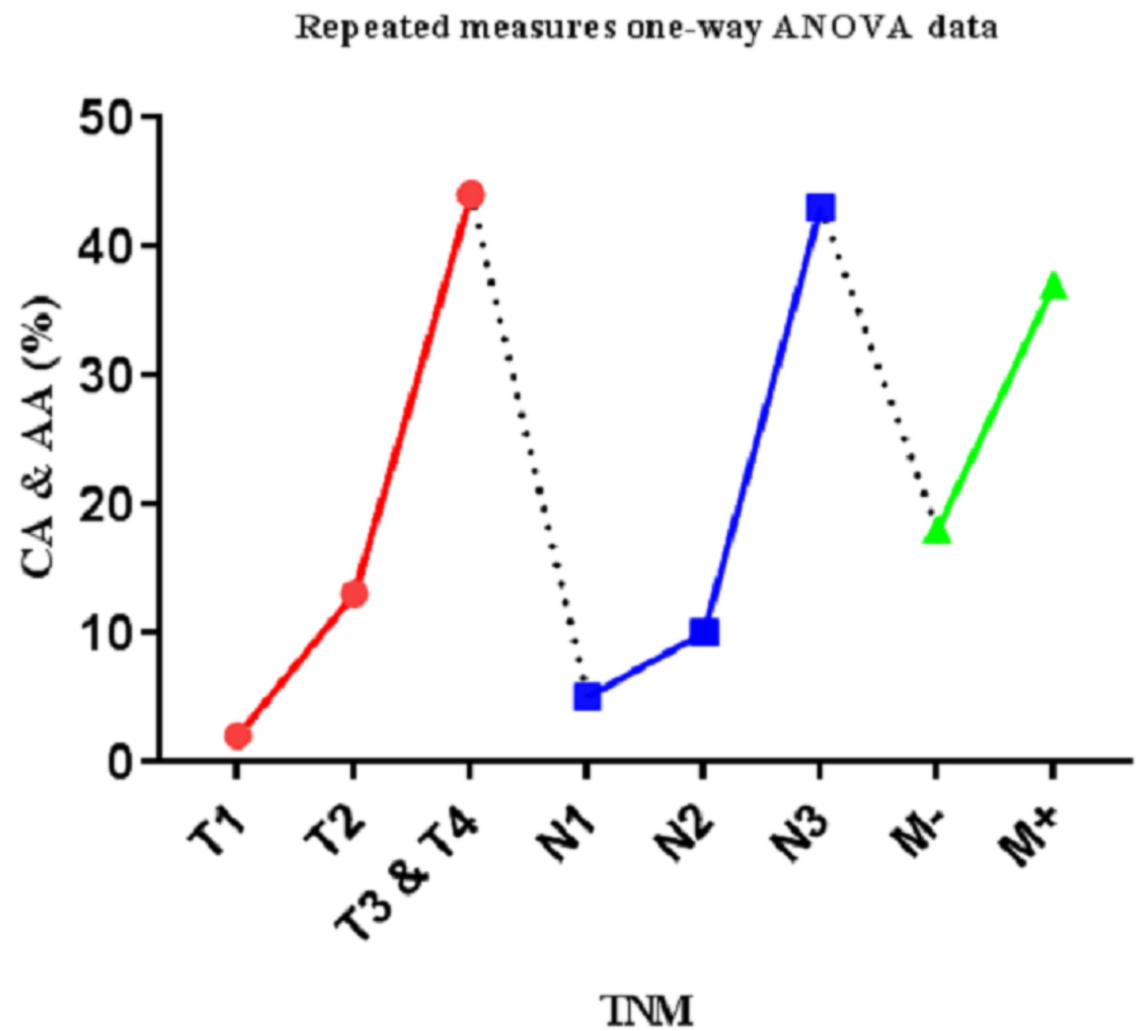
Direct relationship between grade of tumor and metastasis TNM (Tumor, lymph-Node, Metastasis) frequency of C8092A polymorphism of *ERCC1* gene of clinical samples (P<0.05, repeated measures of one-way analysis of variance data

The properties of clinical samples and two SNPs of ERCC1 were evaluated by using GraphPad Prism software version 7.0. In our samples, a higher percentage of CA and AA polymorphisms yielded increased tumor grade (red color, P<0.01, Pearson r analysis, r=0.99, R squared: 0.99), lymph node involvement (blue color, P<0.01, Pearson r analysis, r=0.89, R squared: 0.79) and metastasis (green color, P<0.01, Pearson r analysis, r=0.96, R squared: 0.93) (Figure 3).

All these relationships were statistically significant (P< 0.05, Pearson r analysis, r=0.95, R squared: 0.9037). Also, our study showed that rs11615 SNP did not correlate with the TNM Classification of Malignant Tumors (TNM) stage.

## Discussion

There are several forms of lung cancer. Genomic profiles in NSCLC, performed by comparative genomic hybridization analysis and microarray, have identified gene conflicts and different signaling pathways. Several SNPs have been implicated in genetic susceptibility to cancer. A review study in 2009 examined 1,836 articles from 1986 to 2008 related to genes involved in cancer. Some of these genes are commonly associated with lung cancer [15]. In a study by Zhou et al. in 2004 related to the survival of patients with lung cancer, two main points in ERCC1 including codons 118 and 8092 were identified by studying ERCC1 genotyping [16]. We conducted the present case-control study to investigate the association between two polymorphisms at ERCC1 (118 and 8092) with some clinicopathological features of NSCLC patients such as age, gender, TNM stage, response to therapy and smoking status in the Markazi province of Iran.

A study by Tang et al. in 2014 investigated the existence of SNPs in the replication rate of key genes in lung cancer. Single nucleotide mutations, including polymorphisms of epidermal growth factor receptor (EGFR), KRAS proto-oncogene, GTPase (KRAS), and rearrangements of the ALK receptor tyrosine kinase (ALK) gene, are also involved in the occurrence of low-grade metastatic adenocarcinoma-type lung cancer [17]. In the same year, another meta-analysis study explained that polymorphism of rs11615 in ERCC1 gene correlates with diagnostic applications [18-19]. In our study, the rate of metastasis and involvement of lymph nodes in patients with modified 8092 positions of ERCC1 were 1.46- and 8.2-fold higher, respectively, than the wild-type haplotype. Previous studies have shown that deregulation of ERCC1 is associated with resistance to chemotherapy and ionizing radiation. A study in 2009 mentioned the relationship between ERCC1 and platinum treatment in NSCLC [4]. A study in 2013 investigated the effect of polymorphism in multiple genes on clinical outcomes in patients with colorectal cancer [8]. A study by Eggert et al. in 2015 investigated the relationship of genetic variations in nucleotide binding oligomerization domain containing 1/ caspase recruitment domain4 (NOD1/CARD4) and NOD2/CARD15 genes with a high risk of lung cancer [12]. Results of our study showed that a lower response to chemotherapy is related to a modified haplotype of 8092 rather than a wild-type variant (1.94 x).

The role of leptin receptor gene polymorphism in determining the sensitivity and diagnosis of NSCLC-type lung cancer in the population of affected people in China was also reported by Li et al. in 2012 [20]. Studying the effect of polymorphism on the process of identifying the susceptibility to various cancers, including lung cancer, is very important. In cases where the ERCC1 gene is mutant, the repair system will not be functional. On the other hand, the repair system can be one of the mechanisms of resistance to chemotherapy [16]. As shown in the studies mentioned above, identifying the changes in different codons of the ERCC1 gene is an important issue; consequently, identifying these changes in different populations is necessary.

In studies conducted on various types of cancers, polymorphisms in key genes involved in cancer are directly related to their diagnosis and treatment. Understanding the meaning and concept of polymorphism will lead the doctor to more successful treatment because personalized medicine is performed based on polymorphisms [21]. For example, the relationship between the response to chemotherapy and polymorphism of G4C14 into A4T14 p73 in the exon 2 position in lung cancer was investigated by Li et al. in 2004 [22]. Identifying polymorphism of the angiotensin I converting enzyme (ACE) gene helps determine whether to prescribe the drug Captopril [23]. Detecting polymorphism of the vitamin D receptor (VDR) and forkhead box P3 (FOXP3) genes plays a role in prescribing vitamin D supplements [24]. Diagnosing serotonin transporter (SERT) gene polymorphism is effective in prescribing the drug Fluoxetine [25]. The results of this study align with those mentioned above to help identify cancer and provide successful treatment.

As shown in Table 2, TNM scores in individuals with modified 8092 (CA and AA) were 3.4x, 8.2x and 1.46x higher, respectively, than those for individuals with the CC haplotype. Therefore, surveying a patient’s ERCC1 gene at codon 8092 can likely indicate the patient’s progressive state of NSCLC. The results of this study indicate a significant relationship between the presence of C8092A polymorphism of the ERCC1 gene and the pathological characteristics of patients with NSCLC with partial or no treatment response rates. Also, there is a positive correlation between alteration at this genomics location and persons with NSCLC who smoke cigarettes. However, there was no significant association between clinicopathological characteristics of NSCLC and 118 polymorphism.

The tumorigenesis process of lung cancer is associated with several factors, including cigarette smoke and contaminated air. The samples used were collected from Arak city, which is one of the most contaminated and industrial cities of Iran. Thus, the present study will be additionally important from the epidemiological view, although a larger group should be studied. Our multivariate regression analysis with GraphPad Prism showed a positive correlation between alteration at this genomics location (C8092A) and persons with NSCLC who used to smoke cigarettes.

The ERCC1 gene has a critical role in the repair system. The NER system can be one of the mechanisms of resistance to chemotherapy, so identifying changes in different populations is necessary. The importance of identifying changes in different codons of the ERCC1 gene has been shown in many studies. Therefore, future studies will be necessary to evaluate the expression of this gene in ribonucleic acid (RNA) and protein levels.

## Conclusions

The results of the present study determined a probable correlation between patients’ TNM cancer stage and responses to treatment for patients with C8092A polymorphism of *ERCC1*. Therefore, identifying these changes in different populations can be critical before starting treatment. 
